# Navigating the boundary between ‘normative’ and ‘non‐normative’ collective action: A British case study of the removal of a public statue associated with racism

**DOI:** 10.1111/bjso.70058

**Published:** 2026-03-06

**Authors:** John Dixon, Magi Young, Shelley McKeown, Paul Stenner, Sofia Stathi, Gian Antonio Di Bernardo, Loris Vezzali

**Affiliations:** ^1^ School of Psychology Open University Milton Keynes UK; ^2^ Department of Experimental Psychology University of Oxford Bristol UK; ^3^ School of Human Sciences University of Greenwich London UK; ^4^ Department of Education and Human Sciences University of Modena and Reggio Emilia Reggio Emilia Italy; ^5^ Faculty of Medicine University of Modena and Reggio Emilia Reggio Emilia Italy

**Keywords:** black lives matter, collective action, protest, racism, social change

## Abstract

Psychological research typically distinguishes between *normative* (e.g., peaceful protests, petitions) and *non‐normative* (e.g., property destruction, riots) collective action. This binary framework has proved useful in exploring the psychological factors that shape different forms of collective action. However, recent critiques suggest it oversimplifies the fluid, contested, and context‐dependent nature of collective protest. Our paper develops these critiques through qualitative analysis of walking interview accounts and courtroom transcripts of an event occurring at a 2020 Black Lives Matter rally in the city of Bristol, UK. During this event, a public statue of Edward Colston (1636‐1721), a 17th century slaver, was toppled, defaced, and thrown in the River Avon, and four protestors were subsequently charged with, then acquitted of, criminal damage. Implications for conceptualising and investigating collective action are explored and the importance of recovering the situated meanings and consequences of local understandings of normative and non‐normative action emphasised.

## INTRODUCTION

Psychological research on collective action (CA) has long recognized its diverse forms, often classified into two broad categories—*normative* and *non‐normative*. Normative CA refers to protest activities deemed to adhere to societal laws and standards; peaceful marches, legal sit‐ins or petitions are oft‐cited examples. By contrast, non‐normative CA refers to actions deemed to transgress societal and sometimes legal standards, with examples in the literature including political violence, riots and property destruction. The distinction between these two types of CA has become a focal point for understanding the psychological processes associated with participation, particularly in research on its predictors, moderators and consequences (e.g. Becker & Tausch, 2015; Shuman et al., 2016; Tausch et al., 2011; Li et al., 2024; Teixeira et al., 2019).

Interest in the distinction between normative and non‐normative CA stems both from its applied and theoretical significance. Understanding what leads protesters to transition, for example, from norm‐abiding to norm‐transgressing tactics is believed to have practical implications for addressing problems such as political ‘extremism’, as well as for gauging the effectiveness of different forms of CA in promoting social change (e.g. see Feinberg et al., 2020; Kunst & Obaidi, 2020; Li et al., 2024). Feinberg et al. (2020) explore, for instance, what they call the ‘activist's dilemma’, contending that ‘extreme protest actions’ (e.g. vandalizing animal testing laboratories) may raise public awareness of social issues (e.g. animal rights) yet paradoxically diminish public support (e.g. for anti‐cruelty campaigns). They argue such actions tend to be constructed as ‘immoral’, thereby reducing popular identification with a movement's actions and undermining its cause in the longer term (Teixeira et al., 2019).

On a theoretical level, research indicates that participation in these two forms of CA may follow different motivational and cognitive pathways. Building on Social Identity Theory (SIT; Tajfel & Turner, 1979) and the dual pathway model of CA (van Zomeren et al., 2012a), Becker, Tausch and colleagues have emphasized the distinctive roles played by collective emotions and perceived efficacy in shaping participation in normative versus non‐normative actions (Becker & Tausch, 2015; Tausch et al., 2025). They hold that normative CA is typically motivated by anger at injustice, accompanied by a belief that social change can be achieved. Non‐normative CA, by contrast, is motivated not only by anger but also contempt towards unjust social systems, often accompanied by a belief that change is not achievable through ‘accepted’ channels of resistance.

Shuman et al. (2016) have similarly argued that participants who hold ‘entity theories’ of society—believing that intergroup barriers to progress are fixed and change difficult to achieve—are more likely to feel intensely negative emotions such as hatred. They are thus more ready to transgress societal standards, including via violent protest. This is particularly true for highly identified individuals with strong sense of group belonging and attachment. Conversely, they argue, participants who hold ‘incremental’ theories of the social world, believing that positive change can be accomplished bit by bit, may feel angry at injustice (e.g. their group's perceived historical mistreatment) yet conform to socially ‘accepted’—in Shuman et al.'s terms ‘less extreme’—modes of resistance.

Two issues complicate this line of argument and require further attention. First, psychological research has mainly focused on normative action, leaving comparative evidence relatively fragmented and scant (cf. Saab et al., 2016), though a growing body of research is starting to address this gap (e.g. Carvacho et al., 2023; Li et al., 2024; Stott et al., 2020; Zúñiga et al., 2023 for recent examples). Second, existing evidence is open to varying interpretations. Support for the dual pathway model is summarized by Becker and Tausch (2015) and complemented by Tausch et al.'s (2025) related synthesis of work on ‘radicalization’ and ‘extremism’. In sharp opposition, Uysal et al.'s (2024) systematic review concludes that normative and non‐normative actions share predictors such as identity, efficacy and emotions, challenging the idea that they involve different motivational pathways. Importantly, they also recommend that the binary terminology of ‘normative’ versus ‘non‐normative’ CA be abandoned in favour of recognizing a *continuum* of protest actions that vary in the degree to which they ‘confront’ existing relations of power and inequality.

### Beyond the normative versus non‐normative CA binary

Uysal et al. (2024) suggest that standard definitions of normative and non‐normative protest are problematic in two ways. First, they simplify the nature and consequences of different types of CA within and across contexts, and second, they associate ‘non‐normative’ actions with ‘extremism’ and thus—whether intentionally or not—with unwarranted violations of socially approved values. When researchers speak of actions that breach ‘societal standards’, for example, do they not beg the question of whose standards and whose versions of normativity are being transgressed?

Zúñiga et al. (2023) make a similar point in their analysis of the Aysén movement in Chile, which emerged in response to longstanding grievances about regional underdevelopment and access to services. This movement involved varying kinds of protests, eventually escalating into direct engagements between demonstrators and the police. Drawing on survey and interview data, Zúñiga et al. (2023) found that local participants reported engaging in *both* peaceful and more confrontational tactics and that traditional predictors of CA—group efficacy, anger and identification—were associated with *both* forms of actions, being treated as interconnected elements of a common resistance strategy. Moreover, they found that *positive emotions* like hope, pride and solidarity rather than contempt were critical in sustaining widespread participation in what would conventionally be defined as non‐normative CA. Perhaps most important, they found that a broad coalition of protesters—including categories of people not normally associated with violent resistance, such as mothers with young children—had united to confront police. Activities researchers would ordinarily classify as ‘non‐normative’ (e.g. throwing stones at the police) were conceived by participants as morally legitimate, contextually appropriate and sanctioned by their community. Indeed, they were framed as *self‐defence* in the face of police brutality.

Such findings imply a methodological complexity. What counts as ‘normative’ or ‘non‐normative’ CA is typically defined a priori by CA researchers (see Table 1), using generic scales that enable comparability across studies. Yet such scales arguably disregard how the meanings of CA are locally defined, negotiated and contested, being situated within political struggles over the limits of ‘socially approved’ resistance.

**TABLE 1:** Examples of normative and non‐normative CA as operationalized in psychological research.

	Source
*Normative actions*	
Participating in discussions and meetings	Uluğ et al. (2023)
Making flyers	Tausch et al. (2011)
Signing petitions or complaints	Pauls et al. (2022)
Taking part in a demonstration	Shuman et al. (2016)
Organizing demonstrations	Shuman et al. (2016)
Lobbying or writing to a politician	Pauls et al. (2022)
Willingness to vote in a general election	Tausch et al. (2011)
Discussing a proposed government plan	Shuman et al. (2016)
Cultural performances	Zúñiga et al. (2023)
Donating money to a civil rights organization	Pauls et al. (2022)
Donating to movement organizations	Li et al. (2024)
Forming human chains	Li et al. (2024)
Pot banging	Zúñiga et al. (2023)
*Non‐normative actions*	
Blocking roads	Shuman et al. (2016)
Obstructing government operations and public transportation	Li et al. (2024)
Supporting political violence	Tausch et al. (2011)
Blocking a highway	Tausch et al. (2011)
Throwing rocks or bottles	Shuman et al. (2016)
Attacking politicians or police	Shuman et al. (2016)
Causing property damage	Orazani and Leidner (2019)
Graffitiing government offices	Pauls et al. (2022)
Occupying a public space	Pauls et al. (2022)
Breaking windows and destroying government property	Pauls et al. (2022)
Writing to urge ingroup members to ignore established rules	Wright et al. (1990)
Participating in sit‐ins or unsanctioned occupations	Wright et al. (1990)
Refusing to pay tickets and fines	Shuman et al. (2021)

Building on the elaborated social identity model (ESIM; Drury & Reicher, 2000), contemporary crowd psychology suggests that collective action is rarely either ‘non‐normative’ or ‘normless’; instead, it is governed by the shared identity and internal norms of the group. Crucially, within protesting crowds, such norms tend to be fluid and emergent rather than static. That is, what counts as ‘normative’ often evolves dynamically through interactions between protesters and external authorities (e.g. Drury et al., 2025; Reicher, 1984; Reicher et al., 2004; Stott & Reicher, 1998; Vestergren & Acar, 2023). This process tends to be obscured when observers adopt what Reicher (1984) once labelled an ‘outsider’ perspective, imposing ‘top down’, definitions of normativity. Often espoused by authorities and the popular press, such definitions often also inform the normative versus non‐normative binaries recycled in CA research, as exemplified in Table 1. From this perspective, transgressions of pregiven societal standards (e.g. of public order) are branded as disordered or even ‘violent’ by default, stripping crowd actions of their situated meanings (and indeed overlooking wider debates over what constitutes ‘societal standards’). By contrast, by adopting an ‘insider’ perspective, researchers have shown that crowd behaviour is disciplined and targeted, reflecting a unified effort to realize shared moral values and, critically, unfolding within normative constraints that evolve in relation to contextual dynamics (e.g. Reicher, 1984; Stott et al., 2020; Vestergren & Acar, 2023).

Binary conceptualizations of collective action suffer another limitation: They mask cultural and historical variability. What is deemed ‘non‐normative’ in authoritarian or repressive regimes (e.g. holding a placard) may differ markedly from what is ‘non‐normative’ in more permissive societies, and this may in turn inform the perceived legitimacy of different forms of protest (e.g. see Saavedra & Drury, 2019). As Zúñiga et al. (2023, p. 363) emphasize: ‘The static binary categorization and negative connotation applied to protest violence may arise from the decontextualization of intergroup conflict and protests, which neglects the processes whereby what is seen as normative can change over time’. Indeed, we would argue that the very designation of certain forms of protest as ‘extreme’, ‘violent’ or socially ‘unacceptable’ is an ideological act—an intervention that warrants critical interrogation rather than unreflective adoption of received categories (e.g. those represented in Table 1). Whatever their other advantages, the generic distinctions drawn in social psychological research tend to bypass this reflexive process, reifying what is an essentially contested and evolving definitional boundary.

One response to this problem is to quantify CA using measures adapted and validated under local conditions. This would entail surveying varying local participants (e.g. see also Cocco et al., 2024), including both the general population and relevant activist subgroups. A complementary approach involves qualitative analysis of the frameworks of meaning employed by participants and governing authorities themselves, enabling exploration their local construction and situated consequences. We adopt the latter approach here.

### The present research

We present a case study of the removal of the Edward Colston statue in Bristol, UK, during a Black Lives Matter protest, drawing on walking interviews with protest participants and transcripts from the subsequent criminal trial. The statue's toppling became emblematic of broader debates about historical racism and public memory, culminating in a high‐profile court case in which four participants were charged with, then acquitted of, criminal damage. Our case study thus offers an opportunity to explore how both participants and legal authorities construct the boundary between normative and non‐normative CA in a context where this definition carried profound consequences, both for those involved directly and for the wider Bristol community. Through analysing how these boundaries were negotiated, we advance re‐conceptualizations of protest normativity as emergent and contextually structured.

We adopted a qualitative and rhetorical approach indebted to the work of Billig (1987, 1991; Billig et al., 1988) and embedded in the related traditions of discursive psychology and critical discourse analysis (e.g. Drury, 2002; Gibson, 2015; Potter & Reicher, 1987). This focuses on the role of language and argumentation in everyday thinking and social interaction, emphasizing how rhetorical devices are routinely used by participants in everyday situations to explain, persuade, defend and justify their positions on controversial topics. In doing so, participants not only mobilize ‘sedimented’ (Gibson, 2015) cultural and historical arguments, but also navigate associated contradictions (Billig et al., 1988). That is, they orient to competing historical constructions of controversial issues via a dialectical process that Billig et al. (1988) capture in the concept of ideological dilemma.

The ideological dilemmas approach highlights that public opinion is rarely a coherent or static belief system; instead, it is composed of contradictory ideas that individuals must navigate depending on the specific social context. For example, drawing on interviews with university students, Saavedra and Drury (2019) demonstrate that while participants generally advocate a societal norm that ‘protests should be peaceful’, this norm may conflict with their desire to ‘defend the right to protest’ in certain contexts (e.g. when faced with state repression). Exploring how this dilemma is navigated in practice, they argue, reveals how protesters may reject violence in principle while legitimizing it as ‘self‐defence’ in specific circumstances. In other words, when constructing the ‘acceptability’ of collective action, participants may draw flexibly on historically shared yet contradictory lines of argumentation (for related applications, see Dixon et al., 2006; Iatridis & Kadianaki, 2023; Michos et al., 2021).

Building on this approach, our analysis sought to explore not only how participants *made sense* of a specific event of CA and its aftermath, but also how they *legitimized or challenged* specific forms of CA within a context of local and wider political controversy. In so doing, we sought to move beyond binary distinctions between normative and non‐normative CA, instead highlighting the rhetorically contested and shifting definitional boundaries inherent in their real‐world, situated construction. As we will show, our participants' arguments about the normative acceptability of the Colston protest both drew on and offered (often sophisticated) reflections about the wider ideological dilemmas that surround antiracist protests in the UK and beyond.

## RESEARCH CONTEXT AND METHOD

### Context

On 7 June 2020 following the murder of an African American man, George Floyd, by a white police officer in the United States, a protest took place in Bristol, UK. The protest occurred during the COVID‐19 pandemic and was part of the Black Lives Matter (BLM) movement. Approximately 10,000 participants gathered to challenge systemic racism and police violence. They also ultimately challenged the city's historic connection to slavery through Edward Colston: a 17th‐century slave trader and leading figure in the Royal African Company believed to be responsible for trafficking an estimated 84,000 Africans into slavery. Colston's wealth funded various philanthropic endeavours in Bristol, and his statue—erected in 1895—was originally located in the city centre just off an avenue bearing his name (Figure 2, top panel). The accompanying plaque read: ‘Erected by citizens of Bristol as a memorial of one of the most virtuous and wise sons of their city AD 1895’. The memorialization of Colston, including through the statue, has long been a focal point for local dissent.

**FIGURE 1:**
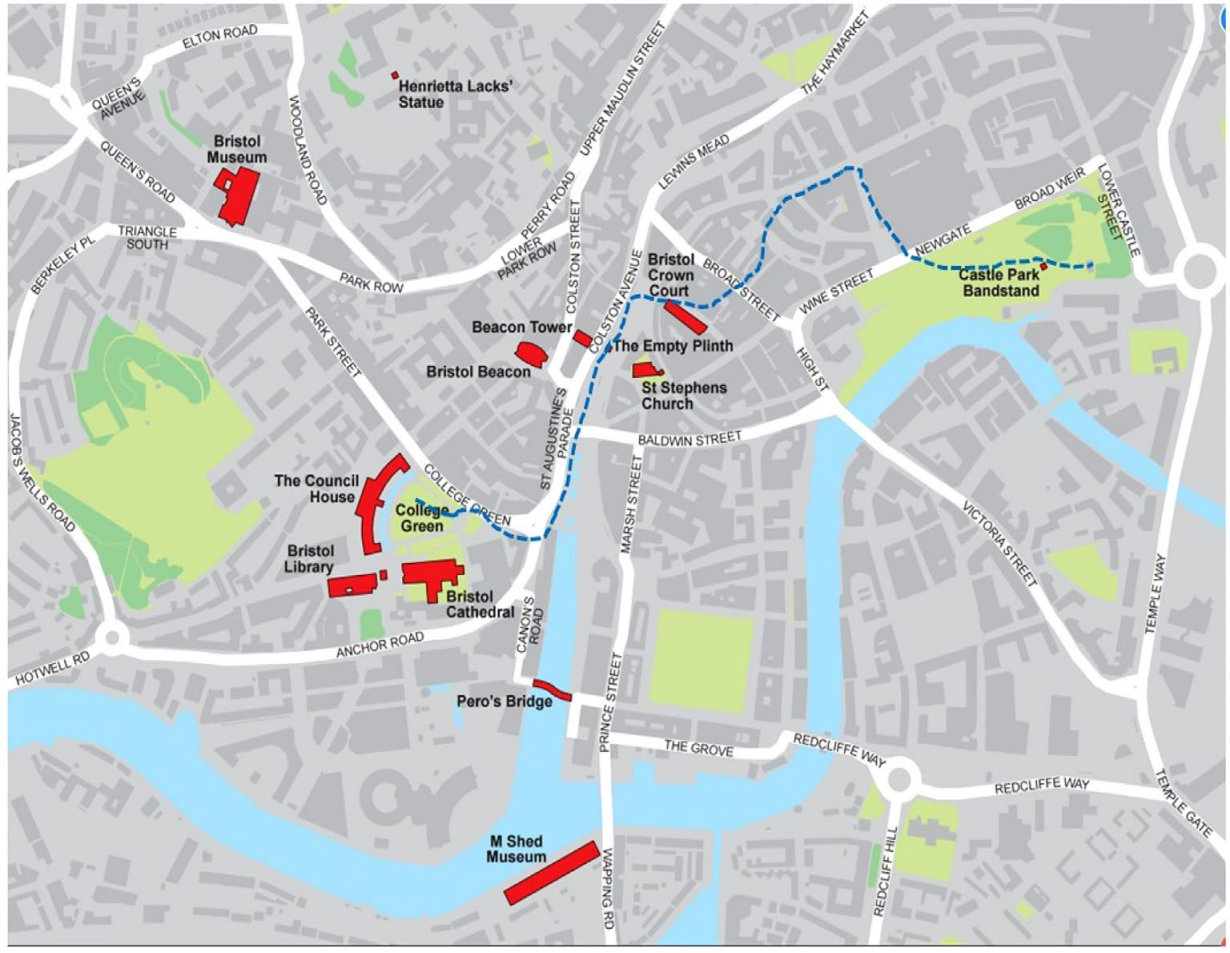
Bristol protest route and key locations. Key sites are indicated in red and the approximate path of the protest in the dashed blue line. College Green was the staging point for the BLM protest march; the empty plinth indicates where Colston's statue originally stood; Pero's bridge—commemorating a former enslaved African—is where the statue was thrown into the harbour; Bristol Crown Court is where the trial of the Colston Four was held; and M Shed is the museum where Colston's statue is currently exhibited.

**FIGURE 2:**
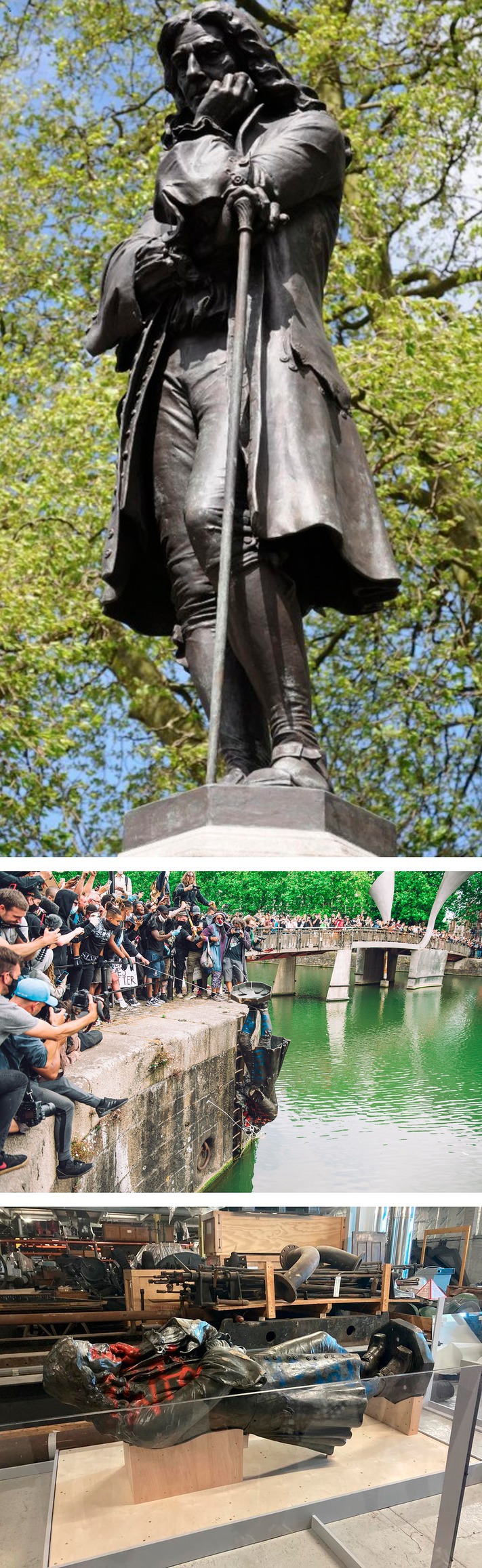
Colston statue in Bristol Note: Top panel: original statue on plinth; Middle panel: being lowered into the river Avon; Bottom panel: museum display. Top panel photo by Philip Halling, licensed under CC‐by‐SA; middle panel, photo by Giulia Spadafora, reproduction rights secured; bottom panel photo by second author, reproduced by permission

The BLM protest began peacefully at College Green, where participants listened to speeches and performed the symbolic act of ‘taking the knee’. Protestors then marched through the city, some stopping at the statue of Colston (see Figures 1 and 2) and the rest proceeding to Castle Park. A small group removed the shroud covering the Colston statue, tied ropes around its neck and toppled it to cheers from some of the crowd. The fallen statue was then rolled through the city's streets and thrown into Bristol harbour (Figure 2, middle panel) near Pero's bridge (Figure 1)—named in honour of Pero Jones, an enslaved African who lived in Bristol in the 18th century.

The statue's toppling sparked intense public debate, with 61% of Bristol residents supporting its removal according to a survey conducted shortly after the event, but a substantive minority opposing it (Miller & Grubb, 2020). Political leaders—including the then UK Prime Minister Boris Johnson and his Home Secretary—condemned the act as criminal damage and public disorder. Four white protesters were prosecuted and, after a trial widely reported in local and national media, the ‘Colston Four’ acquitted. The trial gave international prominence to questions about Britain's colonial past and the role of public monuments in perpetuating systemic racism. In its aftermath, the Council launched a public consultation on the future of the statue and its plinth (Burch‐Brown et al., 2022), and several buildings and schools were renamed.

Retrieved from the river, the statue currently rests in the M Shed's Bristol People Gallery as part of a display about the history of protest in Bristol (Figure 2, bottom panel). A campaign group called ‘The Save our Statues’ has demanded its reinstallation on the original plinth, branding the museum exhibition ‘a celebration of criminal violence and mob rule’ (Gayle, 2021).

### Method

Our study forms part of a broader ethnographic investigation into CA, hope and antiracism. The data that informed the present analysis included walking interviews with protestors and transcripts of trial evidence. The project received ethical approval from the Open University's Human Research Ethics Committee.

#### Walking interviews

Participants (*n* = 22; 10 female, 12 male) were aged from 20 to 70 years. All had participated in the June 2020 protest and were interviewed between July 2022 and October 2023. They were recruited via local activist networks and snowball sampling, and represented a wide range of professions, including community workers, artists, educators, lawyers and students.

The walking interviews were conducted by the second author in and around area mapped in Figure 1. In such ‘walk along’ interviews, data are gathered as both researcher and participant move together through relevant spaces, allowing participants to recall and reflect on experiences in situ (e.g. see Di Masso et al., 2025; Evans & Jones, 2011). This method was selected because it enabled our interviewees to vividly reconnect with key moments during the protest as they retraced the route they and others took through the city. Participants were taken to the empty plinth. Some took us to the harbour and to Castle Park where speeches took place at the end of the protest (Figure 1), depending where they had been on the day of the protest. This allowed them to refer to concrete locations, describe specific movements (e.g. where they stood when the statue fell) and even reenact gestures or actions. The method thus provided rich insights into how they positioned themselves within the protest and how they interpreted their own and others' roles in the CA, including their evaluations of the acceptability of the Colston statue's removal.

Interviews were semi‐structured and lasted between 40 min and 2 h 20 min. Questions were designed to explore participants' experiences during the protest, reflections on their motivations and emotional responses, then and since, evaluations of their own and others' behaviour, and broader interpretations of the implications of the statue's removal and its aftermath. They also explored participants' responses to the statue's toppling and their reflections on the ensuing court case and its verdict.

#### Trial evidence

The trial of four individuals charged with criminal damage to Colston's statue began on 13 December 2021 at Bristol Crown Court. The verdict was delivered on 5 January 2022, the jury finding all four defendants not guilty of criminal damage. Among other things, the trial proceedings addressed the defendants' motivations and justification for their actions. The defence argued broadly that the act was a morally justified and locally supported expression of the legal right to protest, the prosecution that the statue's destruction was an act of criminal damage and public disorder. The trial evidence consisted of full transcripts provided by Bristol Crown Court (Case No: T20210064; comprising in total over 185,000 words), including transcripts of the defence and prosecution's closing arguments and defendants' testimonies and cross‐examinations.

### Analytic approach

We conducted a qualitative analysis grounded in rhetorical and discursive psychology (Billig, 1987; Gibson, 2015; Huma & Potter, 2023; Potter & Wetherell, 1987). Our guiding assumption was that the distinction between ‘normative’ and ‘non‐normative’ collective action is not a fixed behavioural classification that can be expressed in binary terms (e.g. see Table 1 above) but a socially contested boundary. As this boundary is constituted discursively through shifting and contested public argumentation (e.g. moral justifications, appeals to legality, appeals to consensus and historical harm), we argue that it cannot be adequately examined using predefined categories or variable‐based approaches alone. A rhetorical‐discursive methodology is thus not supplementary but theoretically necessary to address the core theme of our work, that is how the boundary between legitimate and illegitimate collective action is *argued into being* rather than an analytic starting point.

Our analytic approach accordingly treated participants' accounts not as transparent reports of internal attitudes but as situated arguments designed to normalize or criticize particular courses of action. As outlined in our introduction, the concept of *ideological dilemmas* (Billig et al., 1988) was used as a sensitizing framework to identify moments where speakers oriented to competing normative values (e.g. respect for the rule of law versus the moral imperative to oppose racism). Analytically, this involved tracing how participants acknowledged, resisted or rebalanced these competing values within single stretches of talk. This allowed us to examine how the boundary between ‘normative’ and ‘non‐normative’ collective action was drawn and rendered consequential in both interview accounts and legal arguments.

In practice, our analysis proceeded through three overlapping stages:

- First, we familiarized ourselves with both the walking interview and courtroom data through repeated readings and formulation of initial coding notes on recurring patterns, themes and linguistic features.
- Second, we identified contrasting patterns of argumentation with respect to formulations of the protest event and its aftermath, tracing their rhetorical organization, contextual variations and discursive functions (see Table 2 for examples identified in the courtroom transcripts). Such arguments often hinged on the construction of the actions of protesters as socially and legally acceptable or unacceptable, ‘normative’ or ‘non‐normative’. This approach allowed us to understand not only *what* participants said but also *how* and *to what discursive ends* they said it.
- Finally, we explored connections between local accounts of the Colston case and wider political discourse on the boundary between licit and illicit forms of CA, that is how participants' accounts oriented to, refracted and reproduced wider ideological traditions and dilemmas (Billig, 1991; Billig et al., 1988; Saavedra & Drury, 2019). As will become apparent, participants themselves often situated the Bristol protest and subsequent trial within its broader historical and political context, orienting to the evolving tension between citizens' right to freedom of protest and the UK state's authority to limit protest in the name of public order. In so doing, as shown below, they also displayed meta‐reflexive awareness of how normative versus non‐normative CA boundary line may shift depending on actors' political orientation and values.

**TABLE 2:** Rhetorical contrasts in the Colston statue trial.

Theme	Defence framing	Prosecution framing
1. The statue as publicly offensive	‘The statue's continued presence was an act of abuse’. ‘It, in my opinion, feels in a sense, a hate crime’	‘There is no evidence that anyone… considered the continued display of the statue itself to be indecent or abusive’
2. Democratic and consensual approval of the protest	‘At the time, I knew we had Bristol behind us, everyone did’. ‘So far as you were aware, how did other people feel about it?’ ‘From everyone I could see, that they wanted it down’	‘10,000 signatures is but a small proportion of the population of this city’. ‘This is the height of arrogance, to presume that you have permission of the people of Bristol’
3. Special case, exceptional circumstances	‘These circumstances are unique to this statue, this city and its people’. ‘There is no evidence any other statue has had over three decades' worth of public outcry’	‘We respectfully say this statue is not unique at all. If we can simply pull down what offends us, regardless of the views of others, then what statues, monuments, buildings and institutions are next, you may ask’
4. Exhaustion of other protest avenues	‘Thousands of people had signed petitions and campaigned nothing was done’. ‘My elders had brought up the petition, they would laugh and say they'd given up’	‘The petition was still up and running when this incident took place’. ‘No defendant made any complaint to any Member of Parliament or the police’
5. Disgust as legitimate moral guide	‘You are entitled to feel horrified, disgusted, revolted’. ‘Sometimes our feelings are so strong you feel viscerally what is right and what is wrong’	‘This trial is not about emotions. This trial is about cold, hard facts, the rule of law’
6. Protestors posed a violent threat to public order	‘His actions cannot possibly be characterized as an act of violence’. ‘It was a deliberate act of solidarity’	‘This cannot on any view be described as peaceful and non‐violent’. ‘The act escalates an atmosphere of peace to one wholly incompatible with that’
7. Legal right to protest	‘Democracy doesn't start and end with the ballot box. The right to protest is part of our history, our democracy, our constitution and the rule of law’	‘The issue here is not whether those in the dock were entitled to freedom of speech but whether they acted unlawfully in purporting to exercise those freedoms’
8. Policing	‘Even the police did nothing, the police watched it… these people are standing there, and they're meant to be doing their job… how can I think it's a crime if I'm doing this and the people who are sent down to be watching us are watching us?’ (Defendant)	‘We made a very tactical decision that to stop people from doing that act may have caused further (disorder)’

As the walking interview and courtroom data featured similar themes and overlapping lines of argumentation, we present an analysis of both data sources together. In order to avoid burdening the reader, however, we have clearly labelled illustrative extracts as deriving either from our ‘walking interviews’ or from the ‘trial transcript’.

## ANALYSIS AND RESULTS

### The statue as moral offence

Before examining how interviewees constructed the nature of CA during the June 2020 protest, we begin by outlining how and why the presence of Colston's statue in Bristol city centre was discursively framed by interviewees as a moral offence, being variously labelled an ‘affront’ (P21), a ‘disgrace’ (P13), an ‘insult’ (P18), an ‘act of violence’ (P12) and a symbol of ‘white supremacy’ (P1). While such characterizations may appear self‐evident, they are important for contextualizing our analysis, illuminating the wider moral landscape within which protest action was understood and justified.

Three elements can be noted here. First, the statue's presence in Bristol city centre was rejected on grounds of Colston's direct involvement in, and profiteering through, the trafficking of African people, his wealth being portrayed as ‘…ill‐gotten gains from enslaving and murdering lots of black people’ (P02). Second, the official portrayal of Colston's legacy was repeatedly criticized. For example, both interview accounts and courtroom records challenged the inscription on the statue's plaque, which venerated Colston as ‘one of the most virtuous and wise sons’ of Bristol. This was rejected as ‘rubbish’ (P15), ‘fake news’ (P18), ‘whitewashing’ (P14) and simply ‘a lie’ (P18). Third, many interview and courtroom accounts narrated the *ongoing* negative impact of the statue on the day‐to‐day lives of Bristol's residents, as illustrated in Extract 1.

**Extract 1: Walking interview, Participant 15**

Interviewer: So, what did you feel, I mean, there's this man who.

Participant: Well, I hated it. Walking past the statue was awful. And I used to do either avoidance or confront it. […] I literally would walk a different way, as opposed to going that way and then going up Corn Street. When I go up Corn Street, I think about all the banks, and all the banks they had to build. Six banks had to be built to put all the money in that was coming from the slave trade. So, it's a continual thing throughout Bristol, really.

Extract 1 shows how the legitimacy of the Colston statue's removal was grounded not only in Colston's historical role in the transatlantic trafficking of enslaved Africans, but also in the ongoing embodied experiences of Bristol's Black residents. As this extract illustrates, the statue was portrayed as imposing a spatial and psychological burden on their everyday navigation of the city. Participant 15, a self‐identified Black woman, describes here how ‘walking past the statue was awful’, prompting strategies of either avoidance or confrontation. This oscillation between avoidance and forced confrontation reflects the enduring affective impact of racialized commemoration in urban space. The participant links this personal discomfort to broader historical injustices, noting that ‘six banks had to be built to put all the money in that was coming from the slave trade’. Here, the statue is not merely a symbolic relic, but a tangible marker of an economy built on slavery, a reminder of unaddressed historical violence. Justification for its removal thus rests simultaneously on appeals to both personal affect and public history—that is as a relief from the routine harms of moving through racialized space as well as a necessary reckoning with the legacy of profiteering.

This kind of argument recurred throughout our walking interviews. Encountering the statue daily was described by participants as reminding Black residents ‘their lives were less important’ (P14), with one participant claiming that it was ‘an act of violence in itself to have people exposed to that and have to look at that’ (P12). Complementary arguments were offered in the legal defence of the Colston Four (as illustrated in Table 2), with lawyers asserting that its continuing presence amounted to a ‘hate crime’ against the people of Bristol. A consistent feature of such arguments was that they conveyed a sense of ‘moral outrage’ (van Zomeren et al., 2012b), appealing to shared values of antiracism. As we will see, this moral framework not only grounded opposition to the statue's presence, but also warranted its removal as a normative, socially sanctioned, course of action.

### Navigating the boundary between normative and non‐normative collective action

We now turn more specifically to constructions of the boundary between normative (licit, socially acceptable) and non‐normative (illicit, socially unacceptable) CA. Three formulations are explored, representing the protest as: (1) expressing the democratic, consensual will of the Bristol people; (2) enacting a largely peaceful, constrained and targeted form of CA that redressed the historical failure of other forms of protest; and (3) expressing higher moral values that transcended narrow legal concerns over public order and criminal damage.

#### Constructing consensus: The statue's removal as ‘The Will of the people’

Both the public debate and the legal proceedings surrounding the toppling of the Colston statue hinged on the issue of consensus. The act was framed in two diametrically opposed ways: as a legitimate expression of the democratic will of the people of Bristol or as an undemocratic act carried out by a minority who unlawfully imposed their will on the city.

For supporters, the statue's removal was framed as the culmination of a long, uniquely Bristolian struggle. In several of our walking interview accounts, its toppling was cast not as an aberration but as a normative act desired and sanctioned by local people. A key rhetorical feature of this framing was the use of metonymic constructions in which ‘Bristol’ itself was personified as the agent of change. One participant, for example, noted ‘this was a Bristol problem, and we gave it a Bristol solution by taking it down’ (P02); another described the protest as ‘so Bristol’, explaining that ‘Bristol has a voice like no other city’. (P15). The defence team in the trial of the Colston Four extended this line of argumentation, arguing that the protesters plausibly felt the statue's toppling to have widespread local approval.

**Extract 2: Trial transcript, Defence lawyer**

Rhian Graham has told you that she honestly and genuinely believed that the people of Bristol wanted the statue removed. She believed the people of Bristol were entitled to consent to its removal and she believed that they would have consented to its removal and to any resultant damage caused by its removal. She believed that on the basis of conversations with fellow Bristolians. She believed that on the basis of the research that she had done, the campaigns against it, the calls for it to be removed, in particular by black councillors and an MP of colour. She believed that on the basis of the petition signed in the days leading up to 7 June march. And she believed it because of the actions of those people, of all walks of life, who congregated around the statue that day. Police officer Julie Haywood estimated that there were in excess of 3000 people around the statue, just under a third of her estimated total of at least 10,000 marchers. She believed because those people around the statue, who joined together, spontaneously, to pull as one on the rope that she had supplied.

Taken from the closing remarks of Blinne Ni Blinne Ní Ghrálaigh, the lawyer who defended Rhian Graham (one of the accused), Extract 2 cites evidence that protest could be reasonably construed as having popular consent. Multiple and accumulating forms of evidence are presented: the defendants' conversations with other residents, the results of public petitions and, crucially, ‘the actions of those people, of all walks of life, who congregated around the statue that day’. The metaphoric image of people ‘who joined together, spontaneously, to pull as one on the rope’ is presented as a direct, unmediated enactment of democratic consent, a manifestation of the city's will. This classic rhetorical ‘appeal to consensus’ (Edwards & Potter, 1992) effectively collapses the diverse population of a city into a unified actor, underplaying internal divisions and imbuing the protestors' actions with a democratic mandate.

By contrast, the prosecution's case was built precisely on dismantling a narrative of popular consensus by re‐framing the statue's removal as undemocratic (and thus ‘non‐normative’ or ‘extreme’). The prosecution repeatedly challenged the defendants' right to act on behalf of the city's population. If supporters of the protest presented it as a ‘Bristol solution to a Bristol problem’, the prosecution instead highlighted protesters' ‘arrogant disregard for the views of others’, defining democratic support narrowly by equating it with formal, institutional, processes. This is exemplified in Extract 3. Here Mr. Hughes—the lead prosecutor—argues that while the council's slow pace was ‘regrettable’, it was nonetheless ‘demonstrative of democracy in action’. Moreover, far from representing a consensually‐accepted action, the statue's removal is portrayed as having been conducted ‘regardless of the views of other Bristolians for democracy to take its course’. Later in proceedings, Hughes similarly appealed to a silent, law‐abiding majority (the ‘vast majority would also disagree with the way it was taken down’), recasting the Colston Four as an unrepresentative faction, who believed ‘…there's only one way. Their way. And their way excludes democracy and embraces unlawful action’.

**Extract 3: Trial transcript, closing speech of prosecuting lawyer**

While of course it's very regrettable that a public body like the council moved slowly in taking steps to rectify what some but not all consider to be the imbalance of historical accuracy, but it's nonetheless demonstrative of democracy in action, and we will come back to that. What were the Defendants' intentions, then, at the time they were undertaking the various acts alleged by the Crown? We say the Defendants' intentions were clear and without issue. The recordings demonstrated these Defendants, the Defendants wanted to pull the statue down, decided to pull the statue down and were going to do it without consideration of the ongoing democratic process, regardless of the views of other Bristolians for democracy to take its course.

#### Failure of ‘proper channels’

**FIGURE 3:**
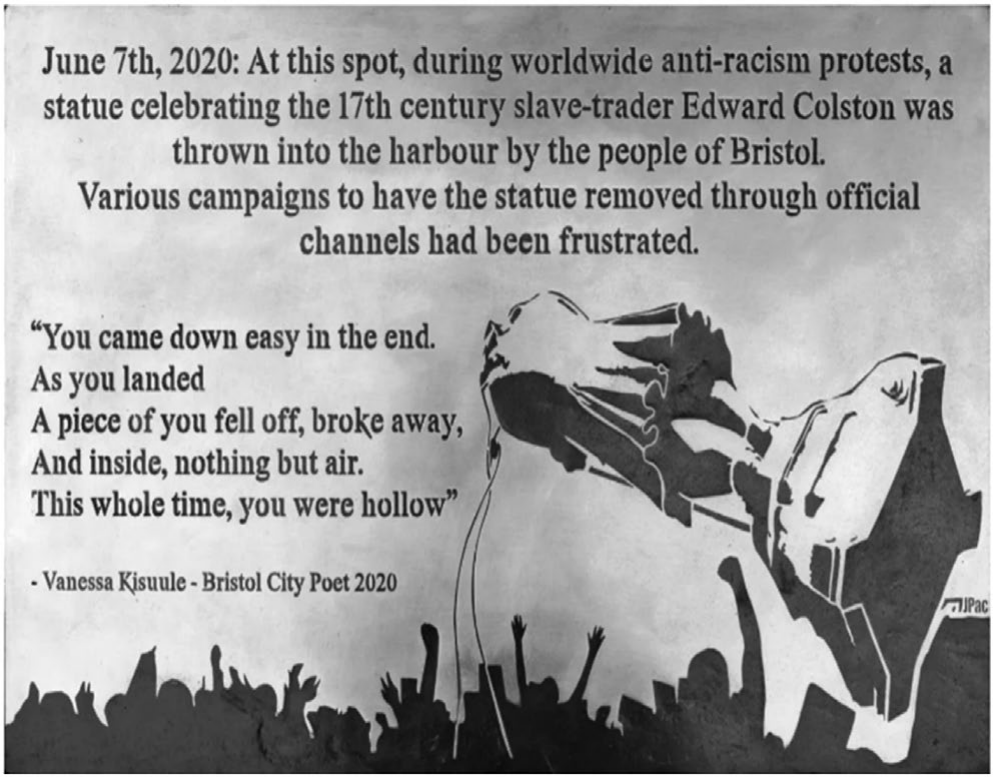
Image of the riverside plaque where Colston's statue was thrown into the harbour.

Figure 3 features a plaque placed at a spot near Pero's Bridge where Colston's statue was thrown into the harbour. It was erected a year after the protest by an anonymous group calling themselves the ‘Guerrilla Historians’ (and is now in the M Shed museum). During his walking interview, one of our participants took the interviewer to this plaque and read out its inscription. This framed the statue's removal not only as part of a wider antiracist struggle by ‘the people of Bristol’, but also a legitimate response to the ineffectiveness of ‘official channels’ of CA.

**Extract 4: Walking interview, Participant 7**

Participant: No, so as I say, my mum's Jamaican, my mum has to walk past that statue, had to walk past that statue every day. And she'd avoid it. She'd avoid it. So, we were taught what Colston did. We knew

Interviewer: Yeah, so you always knew that?

Participant: Yeah, yeah, yeah, completely. And I know that people and the community have commissioned and tried to get it put actually tried to get it taken down. So, through, you know, whether it be, I don't know, there were petitions, there were, you know? There's different means of people trying to remove it in, as some people would say, the correct way. Yeah? But we know that they failed. They know that that they failed, but maybe the council failed them. And didn't see the urgency of removing it.

**Extract 5: Walking interview, Participant 11**

Participant: I know there's been a long, slow debate about what to do about it. And people have been campaigning to get rid of it and the council have been unwilling to do anything about it for a long time. And so, it was a sort of stalemate about what to do. Well, something's got to be done but I'm not sure what. And so, there was that sort of sense of stalemate for a long time. And suddenly it was over, you know? Those young people had just done things. And that it was exhilarating. How to achieve, how to break a stalemate, you know? Be bold and do it.

These extracts together offer a powerful rhetorical justification for the direct action taken against the Colston statue, framing it as a necessary response to prolonged council inaction and a failure of conventional protest methods. The participants also effectively counter the notion that the action was ‘unnecessary’ or ‘extreme’ by appealing to personal experience, community knowledge and moral urgency.

Extract 4 immediately establishes a personal and emotional connection to the issue through his mother's experience (‘my mum has to walk past that statue, had to walk past that statue every day. And she'd avoid it’.), appealing to what we earlier called the ‘embodied experiences’ of residents navigating the city centre. This use of pathos again highlights the everyday discomfort inflicted by the statue's presence on those directly affected by its historical meaning. The speaker's assertion that ‘We were taught what Colston did. We knew’ then builds an ethos of lived experience and inherited knowledge, contrasting this with the implied ignorance of those who might criticize the action. The rhetorical question, ‘maybe the council failed them, then’ shifts blame from the protestors to the council's inaction, implying a moral failing to address a longstanding harm. The final phrase, ‘didn't see the urgency of removing it’, accuses the council of ignoring the issue's gravity and the community's suffering.

Extract 5 introduces the metaphor of a ‘stalemate’ to describe the longstanding inaction regarding the statue, conveying a sense of being stuck, with no progress possible through conventional means. The repeated phrases ‘unwilling to do anything about it for a long time’ and ‘a sort of stalemate about what to do’ emphasize the protracted—and frustrating—nature of the situation. The sudden shift in tone—that is ‘and suddenly it was over, you know? Those young people had just done things. And that it was exhilarating’—creates a stark contrast between the stagnation and the liberating impact of direct action, re‐framing CA as an effective strategy to overcome bureaucratic inertia where conventional methods have failed.

In short, these extracts construct narratives in which the statue's toppling was last resort rather than a rash act, an argument voiced repeatedly by defence lawyers in the trial proceedings and challenged by the prosecuting lawyer (for examples, see Table 2, theme 4 above). They highlight the failure of ‘official channels’, the knowledge and lived experience of those pushing for change, and the justified frustration that led to a decisive, and ultimately effective, breaking of a ‘stalemate’. This kind of rhetorical framing serves an additional ‘defensive’ function (Potter, 1996), countering potential criticisms of the protest as unnecessary or extreme by emphasizing both the history of prior efforts and shared recognition of the Bristol Council's failings. It also orients to the broader issue of what causes a shift from less to more confrontational action, that is conventional approaches had proved ineffective for those demanding the statue's removal.

### A ‘fine line’: The protest as peaceful, constrained and targeted

The prosecuting lawyer in the trial of the Colson Four framed the events as a straightforward case of criminal damage. In so doing, he used terminology that often features in formal academic definitions of non‐normative CA, characterizing the statue toppling as act of ‘violence’, ‘disorder’ and ‘unlawful action’ that was ‘wholly out of proportion’ with acceptable forms of protest and thus a dangerous precedent: ‘You can't go round damaging or destroying things that you don't like, that you hate or disagree with. Therein lies anarchy’.

By contrast, our interviewees generally emphasized the peaceful, orderly and targeted nature of the protest. Perhaps unsurprisingly, the overwhelming majority supported the statue's removal. At the same time, their accounts displayed awareness of the fluid boundary between acceptable and unacceptable forms of protest. Indeed, they revealed how participants oriented to this boundary by challenging external misrepresentations, justifying their perspectives and behaviours, and acknowledging complexity and ambivalence. Given its centrality to our paper's core argument, we provide several examples below.

**Extract 6: Walking interview, Participant 2**

Participant: And you know, what struck me was, you know, this is sort of later on, but later on I started getting messages from friends asking what the hell was going on in Bristol. It had been portrayed in the media as complete chaos and violent, and you know, all this sort of stuff. But I was amazed because if you were there it was so peaceful. It was, you know, there was only, you know, the police stood well back. No one caused any trouble, which was good.

**Extract 7: Walking interview, Participant 12**

Interviewer: Were you worried because you thought it was illegal or was it just because you?

Participant: No, that didn't at any point concern me, not in the slightest. Not in the slightest, it just felt like, ‘This is right, and this is people in the city taking action for how the city should be, how it should feel’, and I didn't know probably at that point about the attempts that had been made to remove the statue through other means, but it just felt, you now, like, with Bristol, we're a city of activism, we're activists, we get things done and when there's something offensive like that, we get it done. And it almost felt like it's being allowed, you know, no‐one's here to stop us, no‐one's here to challenge it, it's almost like it's being condoned by the city.

**Extract 8: Walking interview, Participant 19**

Participant: I think the important thing for me is it's always been about peaceful protesting, because I think if there's any change that's ever going to happen, it's only from that point; as soon as windows start getting smashed—we could save that actually, because we'll talk about that in a minute, yeah.

Interviewer: Well, we'll talk about that, yeah, because that's interesting, I want to hear what you think.

Participant: Yeah, I think it's a very fine line, it's a very fine line, you know, if you're going to start throwing petrol at police officers, you know, it's game over basically, you know, it's kind of whatever cause you're standing up for gets completely pushed aside and it's, like, ‘Oh, you're being violent, you're being aggressive, okay’ that's what becomes the focus.

**Extract 9: Walking interview, Participant 17**

Participant: There was no windows being smashed, there wasn't any sort of other kind of violence to property, it was all focused on the statue basically.

Interviewer: Yeah.

Participant: So yeah, that is a crime, that is a crime, okay, but we have to weigh up the reasons for that action and maybe justify it and stuff.

Across these accounts, peace is positioned as the normative and desirable state of protest. The participant in Extract 6 reflects with incredulity on how the event was later framed: ‘It had been portrayed in the media as complete chaos and violent… But I was amazed because if you were there it was so peaceful’. In so doing, she contrasts the lived experience of insiders who attended the protest with its external misrepresentation, challenging the legitimacy of media narratives. A similar argument is offered in extract 7 in which, again, the protest action is presented consensual, condoned and legal expression of Bristol's activism.

This point is reinforced yet complicated in Extract 8, which begins by articulating the interviewee's personal commitment to peaceful demonstration: ‘it's always been about peaceful protesting, because I think if there's any change that's ever going to happen, it's only from that point’. Such remarks serve to inscribe his actions within culturally endorsed norms of legitimate dissent. They also draw a moral distinction between peaceful and violent protest, for as the participant continues, ‘as soon as windows start getting smashed… it's game over basically’. The repeated phrase ‘a very fine line’ communicates awareness of how protest is mediated, reinterpreted and judged by wider audiences, particularly in contexts where legitimacy is contingent upon normative acceptability. The implication is that the removal of Colston's statue fell on the acceptable side of the line.

While several participants acknowledged that the toppling of the statue potentially constituted a criminal offence, they often framed this act as targeted, symbolically meaningful and, above all, *constrained*. As noted in Extract 9, for example, ‘There was no windows being smashed, there wasn't any sort of other kind of violence to property, it was all focused on the statue basically’. This narrowing of focus is critical. The participant concedes that ‘that is a crime’, but quickly adds, ‘we have to weigh up the reasons for that action and maybe justify it’. Such reasoning foregrounds a sense of proportionality and context, positioning the act not as indiscriminate destruction, but as a morally intelligible response to a specific historical and symbolic grievance. This is very different, as another participant put it, from ‘Smashing up a bus stop and, like, mindless vandalism’ (Participant 01).

Across these accounts, then, participants construct a narrative in which CA is framed as principled, peaceful and reflective of shared civic values. At the same time, they acknowledge the protest's vulnerability to hostile misinterpretation, showing a keen understanding of how legitimacy is discursively produced and contested. The metaphor of a ‘fine line’ captures this tension: a precarious boundary between moral clarity and public condemnation, acceptable and unacceptable protest, that must be carefully managed by those seeking to collectively resist injustice while retaining public sympathy.

The consequences of this definitional struggle to capture the boundary between normative and non‐normative protest—and what or whose definition ultimately takes precedence—were starkly illustrated in the trial proceedings, where the Colston Four faced legal repercussions for actions they and others construed as morally justified, proportionate and socially sanctioned, but which the prosecution framed as criminal damage and disorder. Consider, for example, Extract 10 in which the prosecuting lawyer, Mr. Hughes, interrogates Miss Graham, one of the defendants, about protestors' actions leading up to the statue's toppling.

**Extract 10: Trial transcript, defendant's evidence (cross‐examination by prosecuting lawyer, Mr Hughes)**

Mr Hughes: People were throwing eggs at the statue. So that aspect at the very least had, someone throwing eggs, wasn't exactly peaceful.

Miss Graham: It's peaceful in the sense that people are seeking equality and that man is a man that represents inequality and through his legacy has riven divides between races.

Mr Hughes: Would you describe someone as throwing objects at a statue as peaceful?

Miss Graham: I'd say it comes with good reason.

Mr Hughes: So is the answer yes or no? It is peaceful or it's not. (pause)

Miss Graham: I guess in essence it is not a peaceful act, but it definitely comes with good intention and fighting for the right cause.

This exchange illustrates a rhetorical contestation in which the status of the protest is progressively framed then reframed. Mr. Hughes' questioning works to reclassify the protest as intrinsically non‐peaceful and therefore illegitimate, mobilizing a narrow legal framing of peacefulness as the absence of physical disruption. By contrast, Miss Graham resists this framing by appealing to moral intention, recasting the act not as mere disorder but as justified resistance to racial injustice. The discursive tug‐of‐war centres here not only on how the act is to be defined, but also on which evaluative and normative criteria—legal order versus moral purpose—are accepted.

### Ideological dilemmas in the construction of collective action

Such discursive struggles foreground a wider ideological dilemma faced by liberal democracies: how to reconcile the imperative to limit forms of protest that constitute public disorder with the need to permit—and in some cases protect—disruptive expressions of CA aimed at promoting social justice. As Billig et al. (1988) argued, everyday reasoning is inherently ideological: individuals and communities draw on, contest and reflect the longstanding traditions of public debate that are ‘sedimented’ (Gibson, 2015) within our collectively shared representations. This was powerfully evidenced in our data. Whether in walking interviews or courtroom testimony, protagonists did not simply justify their own or others' actions in the immediate context of relations in Bristol. Rather, they positioned them as part of wider clash of ideological values: between protest and public order, freedom and responsibility, symbolic harm and legal propriety. More than this, they provided sophisticated and reflexive—and sometimes meta‐reflexive—commentary on those same dilemmas, offering competing evaluations of how and where the boundaries of normatively acceptable CA should be drawn.

**Extract 11: Trial transcript, closing speech of prosecuting lawyer**

We in this country are fortunate enough to live in a democracy. We're all entitled to free speech. We're all entitled to express our views without restraint, without rancour and without censorship. We are entitled to protest. We're entitled to demonstrate. We're all free, happily, to articulate our own views. We're entitled to these freedoms provided we undertake them reasonably and within the law. And the issue here is not whether those in the dock were entitled to freedom of speech, entitled to protest, entitled to assemble freely but whether they acted unlawfully in purporting to exercise those freedoms we all take for granted, in pulling down that statue.

**Extract 12: Walking interview, Participant 20**

I understand the argument for a rules‐based order and these kind of things. But then also when I equally think that, you know, what is the violence of bringing down the statue compared to the violence of the Atlantic slave trade, right? And the fallout of that is they're two quite incomparable things. But the way in which that was presented—that this was a really violent act of vandalism and that it spelled the downfall of society, which is kind of seems to be how the Tories were framing it at the time—really, really jarred with what I'd witnessed over the day. It really didn't seem like that at all.

Extract 11, taken from the Prosecution's closing statement, articulates what might be termed a ‘rule‐of‐law liberalism’. It affirms the right to protest while drawing a strict boundary around its lawful enactment. On the surface, the statement affirms democratic freedoms (‘We're all entitled to free speech’), but it simultaneously narrows their legitimate expression to that which occurs ‘within the law’, with the implication that Colston Four's actions fell outside that limit. The effect is to reframe the trial not as an application of justice in the broader moral sense evoked by the defence lawyers, but as a matter of compliance to narrower laws of criminal damage and public disorder.

By way of contrast, consider Extract 12. Here, the protest's meaning is constructed in terms of an argumentative hierarchy that positions moral justice above legal convention. Legality and the need to preserve order and protect public property is acknowledged (‘I understand the argument for a rules‐based order’) but ultimately subordinated to a ‘higher’ imperative that legitimates direct action against statues associated with historical racism, that is ‘the violence of the Atlantic slave trade’. This challenges the moral precedence of property rights. In so doing, the speaker's account also illustrates *meta‐reflexivity*: she does not simply describe competing normative framings but also reflects critically on how such framings are themselves produced in conservative discourse (by ‘The Tories’) and in ways that fundamentally misrepresent the Bristol protest. In other words, the account problematizes the very question that is too often bypassed in CA research: Who gets to draw the line between societally acceptable and unacceptable protest, on what grounds and with what consequences?

## DISCUSSION

Shortly after the removal of Edward Colston's statue, Bristol Mayor Marvin Rees remarked: ‘I cannot condone criminal damage. We need order in the city. But I can't pretend the statue is anything but an affront to me. Not just as a Jamaican heritage man but as a human being’. He subsequently characterized the event as an act of ‘historical poetry’ (Morris, 2020).

The ambivalence inherent in this statement—notably in the tension between civic order and morality—captures the core theoretical tension explored in this paper, which concerns how the ‘societal acceptability’ of collective action is formulated. Specifically, we have contributed to psychological debates concerning the distinction between normative and non‐normative forms of CA, using the toppling of the Colston statue and the subsequent trial of the ‘Colston Four’ as a case study. Our analysis has aimed to move beyond the prevailing tendency to treat this distinction as a fixed, universal binary, defined a priori by researchers and operationalized through standardized behavioural or self‐report items. Instead, we have foregrounded how the definitional boundaries of ‘acceptable’ CA are socially constructed, contested, situated and consequential.

Our findings align with recent critiques (Uysal et al., 2024; Zúñiga et al., 2023) of the field's reliance on generic operationalizations of normative and non‐normative CA. As we have shown, such operationalizations are not always recognized by participants themselves. Nor do they map cleanly onto locally situated accounts of protest. In the Colston case, protestors framed the toppling of the statue not as an act of ‘extremism’ or ‘criminal damage’, but as a constrained, morally principled, democratic and long overdue form of redress. Conversely, official definitions of legality and public order—and reports in some sections of the media—attempted to strip the protest of moral legitimacy, recasting it as a threat to public order and an incitement to ‘vandalism’.

Our analysis contributes to the field in several ways. First, it critically extends perspectives on CA (e.g. Becker & Tausch, 2015; van Zomeren et al., 2012b). It suggests that the boundary between normative and non‐normative action cannot be understood solely through predefined categories that are then linked to individual psychological motivations (e.g. levels of anger, contempt or efficacy), for what counts as ‘normative’ protest is not simply a matter of breaching or respecting pre‐existing ‘societal standards’. Rather, normativity itself must be conceptualized as a social and discursive achievement, continuously negotiated through contestation between protestors, public institutions and legal authorities. Studying this process, we argue, should be a central objective for social psychology.

Second, while our paper does not either challenge or validate a specific theoretical model, such as the dual process model developed by Tausch, Becker and colleagues, it does indicate how a more nuanced understanding of the normative vs. non‐normative distinction might enrich and qualify such models. Tausch et al. (2011) themselves have acknowledged a potential ‘middle category’ of protest, including non‐normative but non‐violent actions (e.g. street blocking; see also Shuman et al., 2021). This aligns with the Colston case, where participants perceived a ‘fine line’ between acceptable and unacceptable protest, differentiating the statue's toppling from ‘mindless vandalism’. Greater attention to such variations could inform more nuanced, contextually specified, theories of CA, helping to reconcile mixed evidence for dual process models (e.g. Uysal et al., 2024).

Relatedly, our paper highlights how *moral reasoning* can be deeply implicated in the normative/non‐normative CA distinction, supporting calls to make this a more central component of theory development (van Zomeren et al., 2018). In the Colston case, protesters weighed the moral offence of the statue—understood as a symbol of historical violence—against the action of toppling it. This moral framework allowed them to justify their actions as a legitimate response to a violation of fundamental values, even when such actions technically fell outside of some conventional definitions of legal or societal norms. The legitimacy of CA, then, was not determined solely by its adherence to pre‐existing social standards, but also by its alignment with a community's core moral beliefs, which in turn became a potent motivator for action (van Zomeren et al., 2018).

Our analysis also underscores the significance of the elaborated social identity model (ESIM) in understanding how collective action is normatively constrained rather than inherently ‘disorderly’. In our Bristol case study, the limits of the protest were not fixed a priori but emerged, at least in part, through interactions with authorities as well as between protesters. As predicted by the ESIM (Drury & Reicher, 2000), what was deemed ‘normative’ on the day of the protest was shaped both by participants' identity‐relevant norms and by police behaviour. Previous research has often focused on how police aggression can encourage a normative shift towards reciprocal escalation (e.g. Stott et al., 2020). In our case study, by contrast, police *restraint* on the day indicated to some protesters that the statues' removal, while not condoned by the officers present, fell within acceptable protest limits^1^ (e.g. Extract 6). Furthermore, during the subsequent trial, the ‘hands off’ nature of policing was explicitly cited as a factor that allowed the protest to remain *peaceful and targeted*, preventing an escalation into violence (see Table 2, theme 8). This reinforces the argument that normative boundaries in events of collective action should be seen as dynamic, evolving and co‐constructed in nature rather than pregiven (Vestergren & Acar, 2023).

We also emphasize that struggles to define what counts as acceptable CA are not specific to the Colston event. As Billig (1987; Billig et al., 1988) have argued, when people debate controversial political actions or events, they routinely draw upon ideological traditions and culturally familiar lines of reasoning. In so doing, they must navigate ideological dilemmas—recurring and conflicting themes in the public discourses that structure everyday reasoning. This includes the tension between upholding public order and the rule of law on the one hand and the freedom of protest on the other.

Both sides of this dilemma are valorized in liberal‐democratic societies. As such, our participants' accounts frequently reflected efforts to reconcile competing normative demands, ultimately in ways that carried material consequences for the Colston Four. It is worth noting that the Colston trial was not the first such case to end in acquittal. Over recent decades, British juries have repeatedly declined to convict protestors charged with criminal damage or public disorder, including climate activists, anti‐arms campaigners and others who framed their actions in moral and political terms.^2^ Such verdicts underscore our broader point: what counts as socially approved protest is not fixed by statute, settled by government or anchored in consensual norms. Rather, it is continually negotiated (and sometimes rejected) by lay publics, protesters, commentators, state functionaries, lawyers and jurors. From this perspective, the acquittal of the Colston Four is one instance in which received definitions of normativity were redefined through legal and moral deliberation.

### Limitations and future directions

As with all case studies, ours is both enriched and restricted by its specificity. The Colston protest was a localized event within a particular cultural, historical and political context. Although its themes resonate with broader movements (e.g. BLM, the decolonization of public monuments; Badilla & Aguilera, 2021), caution is warranted in generalizing from this case to others. Moreover, we have focused mainly on the perspectives of protest participants rather than opponents. While our analysis captures the rhetorical contestation of normativity, it does not fully document the perspectives of those who supported the prosecution of Colston Four or opposed the statue's removal. Similarly, although we drew on official transcripts, our analysis does not capture the full range of legal arguments offered at trial nor, a fortiori, explain why the jury acquitted.

Our analysis points to several avenues for further research. First, we suggest that the study of CA—particularly work grounded in dual pathway models—should attend more closely to the interpretive practices through which the nature of protests is constituted by participants in context.^3^ We note here that the analysis of data on authorities' perspectives on protest violence is comparatively rare in psychological research, yet potentially revealing (see also Saavedra & Drury, 2024). Our case study shows, for example, that even within dominant institutions of state (e.g. the social justice system) official definitions of what counts as ‘non‐normative CA’ may be shifting and contested. The Colston Four were ultimately acquitted as result of this dynamic process, with one institutional version of ‘societally accepted’ CA ultimately trumping another.

Second and closely related, our work raises methodological questions about the widespread use of standardized rating scales to distinguish between normative and non‐normative protest. Whatever their advantages, such tools risk treating these categories as universal and immutable, when in fact they are contextually specific, malleable and imbued with contested social meanings. On the one hand, our analysis underscores the continuing value of qualitative methods in CA research. On the other, it supports Cocco et al.'s (2024) call to develop ‘…more contextualized and community‐driven measures of CA, which account for the local meanings and practices of activism and involve participants in defining what constitutes action within their socio‐political environment’ (p. 170). This move is broadly consistent with Uysal et al.'s (2024) proposal to replace the normative versus non‐normative dichotomy with a framework in which CA is classified as falling along a more to less *confrontational* continuum. However, while we see this as a useful intervention, we worry it is open to the same processes of decontextualization and reification as the normative versus non‐normative binary. The degree to which CA is evaluated ‘confrontational’, after all, is itself fluid, situated, rhetorical and contestable. Our main point in the present paper, then, is that researchers need to adopt methods and concepts that can explore varying constructions of CA and trace the situated effects of constructing its normativity in varying ways.

Third, as noted already, future research might also explore how the tension between normative and non‐normative CA relates to moral reasoning (van Zomeren et al., 2012b; 2018). This could examine, for instance, how different ‘moral orders’ clash. In the Colston case, moral discourse about property law and public order conflicted with discourse about antiracism and historical redress; indeed, the trial became a site where these ‘normative pluralisms’ contended for legitimacy. This approach contrasts, for example, with Feinberg et al.'s (2020) argument that ‘extreme’ protest simply diminishes public support. To the contrary, our data suggest that actions conventionally treated as non‐normative (e.g. statue toppling) can, under specific conditions, be morally justified by participants and wider publics. The meaning of protest ‘immorality’ is not fixed. Rather, complicating the so‐called ‘activist's dilemma’, it is dilemmatic in the fuller sense of the term, often operating in the ‘grey area’ between legitimacy and illegitimacy (cf. Siraz et al., 2023) and implicating social struggles to define the moral grounds of CA.

Fourth, we would highlight how the ideological diversity of protest further destabilizes the normative/non‐normative binary. While the Colston toppling was framed as a targeted act of antiracist resistance, Aguilera and Badilla's (2022) analysis of *memorialicidio* in Chile reveals how conservative actors may seek to erase the political legacies of marginalized groups through the removal or defacement of public monuments. This comparison suggests that the struggle to define ‘socially acceptable’ collective action (CA) is an across‐the‐spectrum phenomenon. It invites researchers to move beyond the study of progressive movements to understand how the boundaries of normativity may be tested across disparate forms of CA.

Finally, future studies could also investigate how support for CA varies depending on its normative framings (e.g. public safety vs. historical injustice). This would extend van Zomeren et al.'s (2018) work on moral conviction by exploring how moral framings influence attributions of protest intentions and perceptions of the legitimacy of ‘non‐normative’ CA. The acquittal of the Colston Four, and similar cases, suggests that juries, when faced with a ‘greater crime’, may view protest actions that technically violate the law as morally justified. In historical hindsight, many successful social movements gained legitimacy precisely through their ability to reframe actions once castigated as unacceptable as both necessary and morally justified, thereby transforming public understandings of what constitutes legitimate collective action.

## AUTHOR CONTRIBUTIONS

**John Dixon:** Conceptualization; data curation; formal analysis; visualization; writing – original draft; methodology; investigation; writing – review and editing; funding acquisition. **Magi Young:** Data curation; formal analysis; visualization; writing – original draft; methodology; project administration; writing – review and editing. **Shelley McKeown:** Conceptualization; writing – review and editing; funding acquisition. **Paul Stenner:** Formal analysis; writing – original draft; writing – review and editing; conceptualization. **Sofia Stathi:** Conceptualization; writing – review and editing; funding acquisition. **Gian Antonio Di Bernardo:** Conceptualization; writing – review and editing. **Loris Vezzali:** Conceptualization; writing – review and editing.

## FUNDING INFORMATION

This work was supported by funding awarded by the British Academy (Ref. SRG2021\210141).

## CONFLICT OF INTEREST STATEMENT

Co‐author Shelley McKeown is a co‐Editor of the *British Journal of Social Psychology*.

## Data Availability

The qualitative data (interview transcripts and field notes) supporting the findings of this study are not publicly available due to ethical restrictions concerning participant privacy and the sensitive nature of the topics discussed. Anonymised excerpts are included within the manuscript. Access to the full courtroom transcripts used in this analysis is subject to the permissions of the Bristol Crown Court (Case No: T20210064).

## Footnotes

^1^
One protester highlighted police officers' seemingly *deliberate* inattention to the statue's removal: ‘…it was just hilarious because it was just—Oh sorry, yeah. It was just everywhere. They were looking everywhere except for at the statue passing by them. It's just like, I remember looking, I remember looking at and it's just like ‘They don't give a shit’. They're not on their walkie talkies, they're not doing … they are looking everywhere else’.

^2^
British juries have repeatedly acquitted activists who took direct action against the arms trade, for example, judging their actions justifiable in light of the harms they sought to prevent (e.g. see Double Down News, 2025). In 1996, four women who sabotaged UK warplanes bound for Indonesia were found not guilty due to atrocities in East Timor. In 2003, activists disrupting fuel tankers for US planes to Iraq were acquitted—one defended by Keir Starmer, Current UK prime minister, now criticized for moves to label peaceful protesters as terrorists. In 2017, those who tried to disarm Typhoon jets headed to Saudi Arabia were also cleared, given concerns over war crimes in Yemen. Most recently, in 2024, a jury acquitted Palestine Action for targeting a weapons firm. In the trial of the Colston Four, the defence lawyers cited this wider history of juror support for activism in the public and moral good (e.g. the suffragette struggle).

^3^
Ironically, this ‘future direction’ was flagged in Reicher's (1984) classic study of collective action in Bristol over 40 years ago.

## References

[bjso70058-bib-0001] Aguilera, C. , & Badilla, M. (2022). Human rights memorials in turmoil: Antagonistic memories in contemporary Chile. Political Geography, 98, 102731. 10.1016/j.polgeo.2022.102731

[bjso70058-bib-0002] Badilla, M. , & Aguilera, C. (2021). The 2019–2020 Chilean anti‐neoliberal uprising: A catalyst for decolonial de‐monumentalization. Memory Studies, 14(6), 1226–1240. 10.1177/17506980211054305

[bjso70058-bib-0003] Becker, J. C. , & Tausch, N. (2015). A dynamic model of engagement in normative and non‐normative collective action: Psychological antecedents, consequences, and barriers. European Review of Social Psychology, 26(1), 43–92.

[bjso70058-bib-0004] Billig, M. (1987). Arguing and thinking: A rhetorical approach to social psychology. Cambridge University Press.

[bjso70058-bib-0005] Billig, M. (1991). Ideology and opinions: Studies in rhetorical psychology. Sage.

[bjso70058-bib-0006] Billig, M. , Condor, S. , Edwards, D. , Gane, M. , Middleton, D. , & Radley, A. (1988). Ideological dilemmas: A social psychology of everyday thinking. Sage.

[bjso70058-bib-0007] Burch‐Brown, J. , Cole, T. , Burton, E. , Costley, N. , Poole, S. , Sobers, S. , & Tincknell, E. (2022). The Colston Statue: What next? We Are Bristol History Commission Short Report. Bridging Histories. www.bridginghistories.com/heritage‐resources

[bjso70058-bib-0008] Carvacho, H. , González, R. , Cheyre, M. , Rocha, C. , Cornejo, M. , Jiménez‐Moya, G. , Manzi, J. , Álvarez‐Dezerega, C. , Álvarez, B. , Castro, D. , Varela, M. , Valdenegro, D. , Drury, J. , & Livingstone, A. (2023). When social movements fail or succeed: Social psychological consequences of a collective action's outcome. Frontiers in Psychology, 14, 1155950. 10.3389/fpsyg.2023.1155950 37179879 PMC10172655

[bjso70058-bib-0009] Cocco, V. M. , Vezzali, L. , Stathi, S. , Di Bernardo, G. A. , & Dovidio, J. F. (2024). Mobilizing or sedative effects? A narrative review of the association between intergroup contact and collective action among advantaged and disadvantaged groups. Personality and Social Psychology Review, 28(2), 119–180. 10.1177/10888683231203141 37864514 PMC11010580

[bjso70058-bib-0010] Di Masso, A. , Buser, M. , & Palmadottir, H. (2025). What is so good about walking interviews? Expanding the geo‐indexical virtues of making meaning on the move. Qualitative Research in Psychology, 22(1), 1–21. 10.1080/14780887.2025.2459704

[bjso70058-bib-0011] Dixon, J. , Levine, M. , & McAuley, R. (2006). Locating impropriety: Street drinking, moral order, and the ideological dilemma of public space. Political Psychology, 27, 170–190. 10.1111/j.1467-9221.2006.00002.x

[bjso70058-bib-0012] Double Down News . (2025). Sharing This Video Could Get You 14 Years in Prison [Video]. YouTube.

[bjso70058-bib-0013] Drury, J. (2002). “When the mobs are looking for witches to burn, nobody's safe”: Talking about the reactionary crowd. Discourse & Society, 13(1), 41–73. 10.1177/0957926502013001003

[bjso70058-bib-0014] Drury, J. , Ball, R. , & Poole, S. (2025). Solidarity riots in the diffusion of collective action: Doing historical research to develop theory in social psychology. British Journal of Social Psychology, 64, e12870. 10.1111/bjso.12870 40013956 PMC11867052

[bjso70058-bib-0101] Drury, J. , & Reicher, S. (2000). Collective action and psychological change: The emergence of new social identities. British Journal of Social Psychology, 39(4), 579–604. 10.1348/014466600164642 11190686

[bjso70058-bib-0015] Edwards, D. , & Potter, J. (1992). Discursive psychology. Sage.

[bjso70058-bib-0016] Evans, J. , & Jones, P. (2011). The walking interview: Methodology, mobility and place. Applied Geography, 31(2), 849–858. 10.1016/j.apgeog.2010.09.005

[bjso70058-bib-0017] Feinberg, M. , Willer, R. , & Kovacheff, C. (2020). The activist's dilemma: Extreme protest actions reduce popular support for social movements. Journal of Personality and Social Psychology, 118(1), 15–41. 10.1037/pspi0000230 31928025

[bjso70058-bib-0018] Gayle, D. (2021). Campaigners try to block Edward Colston display at Bristol museum. *The Guardian*, June 7. https://www.theguardian.com/uk‐news/2021/jun/07/campaigners‐try‐to‐block‐edward‐colston‐display‐at‐bristol‐museum

[bjso70058-bib-0019] Gibson, S. (2015). From representations to representing: On social representations and discursive‐rhetorical psychology. In G. Sammut , E. Andreouli , G. Gaskell , & J. Valsiner (Eds.), The Cambridge handbook of social representations (pp. 210–223). Cambridge University Press.

[bjso70058-bib-0020] Huma, B.‐R. , & Potter, J. (2023). Discursive psychology. In M. Handford & J. P. Gee (Eds.), Routledge handbook of discourse analysis (2nd ed., pp. 53–66). Routledge. 10.4324/9781003035244-6

[bjso70058-bib-0021] Iatridis, T. , & Kadianaki, I. (2023). Constructions of difference in lay talk about diversity: Ideological dilemmas, antiracism and implications for identity. British Journal of Social Psychology, 62(3), 1271–1284. 10.1111/bjso.12631 36756863

[bjso70058-bib-0023] Kunst, J. R. , & Obaidi, M. (2020). Understanding violent extremism in the 21st century: The (re)emerging role of relative deprivation. Current Opinion in Psychology, 35, 55–59. 10.1016/j.copsyc.2020.03.010 32344297

[bjso70058-bib-0024] Li, M. , Adra, A. , Yuen, S. , Salfate, S. V. , Chan, K.‐M. , & Baumert, A. (2024). Understanding non‐normative civil resistance under repression: Evidence from Hong Kong and Chile. Political Psychology, 45(3), 493–515. 10.1111/pops.12933

[bjso70058-bib-0026] Michos, I. , Figgou, L. , & Bozatzis, N. (2021). Constructions of LGBTQI+ rights and claims in lay discourse in Greece: Liberal dilemmas and sexual citizenship boundaries. Journal of Community & Applied Social Psychology, 31(6), 768–781. 10.1002/casp.2538

[bjso70058-bib-0027] Miller, C. , & Grubb, S. (2020). Bristol has spoken ‐ and most people are glad the Colston statue was pulled down. *Bristol Live*, 13 June 2020. https://www.bristolpost.co.uk/news/bristol‐news/bristol‐spoken‐most‐people‐glad‐4217554

[bjso70058-bib-0028] Morris, S. (2020). Bristol mayor: Colston statue removal was act of “historical poetry”. The Guardian, June 13. https://www.theguardian.com/uk‐news/2020/jun/13/bristol‐mayor‐colston‐statue‐removal‐was‐act‐of‐historical‐poetry

[bjso70058-bib-0029] Orazani, S. N. , & Leidner, B. (2019). The power of nonviolence: Confirming and explaining the success of nonviolent (rather than violent) political movements. European Journal of Social Psychology, 49(4), 688–704. 10.1002/ejsp.2526

[bjso70058-bib-0030] Pauls, I. L. , Shuman, E. , Saguy, T. , & Halperin, E. (2022). Does crossing a moral line justify collective means? Explaining how a perceived moral violation triggers normative and nonnormative forms of collective action. European Journal of Social Psychology, 52(1), 105–123. 10.1002/ejsp.2818

[bjso70058-bib-0031] Potter, J. (1996). Representing reality: Discourse, rhetoric and social construction. Sage.

[bjso70058-bib-0032] Potter, J. , & Reicher, S. (1987). Discourses of community and conflict: The organization of social categories in accounts of a ‘riot’. British Journal of Social Psychology, 26(1), 25–40. 10.1111/j.2044-8309.1987.tb00758.x

[bjso70058-bib-0033] Potter, J. , & Wetherell, M. (1987). Discourse and social psychology: Beyond attitudes and behaviour. Sage.

[bjso70058-bib-0034] Reicher, S. , Stott, C. , Cronin, P. , & Adang, O. (2004). An integrated approach to crowd psychology and public order policing. Policing, 27(4), 558–572. 10.1108/13639510410566271

[bjso70058-bib-0035] Reicher, S. D. (1984). The St. Pauls' riot: An explanation of the limits of crowd action in terms of a social identity model. European Journal of Social Psychology, 14(1), 1–21. 10.1002/ejsp.2420140102

[bjso70058-bib-0036] Saab, R. , Spears, R. , Tausch, N. , & Sasse, J. (2016). Predicting aggressive collective action based on the efficacy of peaceful and aggressive actions. European Journal of Social Psychology, 46(5), 529–543. 10.1002/ejsp.2193

[bjso70058-bib-0037] Saavedra, P. , & Drury, J. (2019). Beyond peaceful protest: When non‐participants support violence against the police. *PsyArXiv*. 10.31234/osf.io/rm7jg

[bjso70058-bib-0038] Saavedra, P. , & Drury, J. (2024). Understanding protest violence: From protesters' to non‐participants' support for protest violence. In J. R. Vollhardt & F. Bou Zeineddine (Eds.), Resistance to repression and violence: Global psychological perspectives (pp. 202–225). Oxford University Press. 10.1093/9780197687703.003.0010

[bjso70058-bib-0039] Shuman, E. , Cohen‐Chen, S. , Hirsch‐Hoefler, S. , & Halperin, E. (2016). Explaining normative versus nonnormative action: The role of implicit theories. Political Psychology, 37, 835–852. 10.1111/pops.12325

[bjso70058-bib-0040] Shuman, E. , Saguy, T. , van Zomeren, M. , & Halperin, E. (2021). Disrupting the system constructively: The effect of nonnormative nonviolent collective action on advantaged group members' support for social change. Journal of Personality and Social Psychology, 121(4), 819–841. 10.1037/pspi0000336 32790473

[bjso70058-bib-0041] Siraz, S. S. , Claes, B. , De Castro, J. O. , & Vaara, E. (2023). Theorizing the grey area between legitimacy and illegitimacy. Journal of Management Studies, 60(4), 924–959. 10.1111/joms.12901

[bjso70058-bib-0042] Stott, C. , Ho, L. , Chan, Y. T. , Kyprianides, A. , & Saavedra, P. (2020). Patterns of ‘disorder’ during the 2019 protests in Hong Kong: Policing, social identity, intergroup dynamics, and radicalization. Policing: A Journal of Policy and Practice, 14, 814–835. 10.1093/police/paaa073

[bjso70058-bib-0043] Stott, C. , & Reicher, S. (1998). How conflict escalates: The inter‐group dynamics of collective football crowd ‘violence’. Sociology, 32(2), 353–377. 10.1177/0038038598032002007

[bjso70058-bib-0044] Tajfel, H. , & Turner, J. (1979). An integrative theory of intergroup conflict. In W. G. Austin & S. Worchel (Eds.), The social psychology of intergroup relations (pp. 33–47). Brooks/Cole.

[bjso70058-bib-0045] Tausch, N. , Becker, J. C. , Spears, R. , Christ, O. , Saab, R. , Singh, P. , & Siddiqui, R. N. (2011). Explaining radical group behavior: Developing emotion and efficacy routes to normative and nonnormative collective action. Journal of Personality and Social Psychology, 101, 129–148. 10.1037/a0022728 21500925

[bjso70058-bib-0046] Tausch, N. , Bode, S. , & Halperin, E. (2025). Emotions in violent extremism. In M. Obaidi & J. Kunst (Eds.), The Cambridge handbook of violent extremism. Cambridge University Press.

[bjso70058-bib-0047] Teixeira, C. P. , Spears, R. , & Yzerbyt, V. Y. (2019). Is Martin Luther king or Malcom X the more acceptable face of protest? High‐status groups' reactions to low‐status groups' collective action. Journal of Personality and Social Psychology, 118, 919–944. 10.1037/pspi0000195 31169387

[bjso70058-bib-0102] Uluğ, Ö. M. , Chayinska, M. , & Tropp, L. R. (2023). Does witnessing gender discrimination predict women's collective action intentions for gender justice? Examining the moderating role of perceived female support. Journal of Community & Applied Social Psychology, 33(2), 501–518. 10.1348/014466600164642

[bjso70058-bib-0048] Uysal, M. S. , Saavedra, P. , & Drury, J. (2024). Beyond normative and non‐normative: A systematic review on predictors of confrontational collective action. British Journal of Social Psychology, 63, 1385–1409. 38390962 10.1111/bjso.12735

[bjso70058-bib-0049] van Zomeren, M. , Kutlaca, M. , & Turner‐Zwinkels, F. (2018). Integrating who “we” are with what “we” (will not) stand for: A further extension of the social identity model of collective action. European Review of Social Psychology, 29(1), 122–160. 10.1080/10463283.2018.1479347

[bjso70058-bib-0050] van Zomeren, M. , Leach, C. W. , & Spears, R. (2012a). Protesters as “passionate economists”: A dynamic dual pathway model of approach coping with collective disadvantage. Personality and Social Psychology Review, 16(2), 180–199. 10.1177/1088868311430835 22241796

[bjso70058-bib-0051] van Zomeren, M. , Postmes, T. , & Spears, R. (2012b). On conviction's collective consequences: Integrating moral conviction with the social identity model of collective action. British Journal of Social Psychology, 51(1), 52–71. 10.1111/j.2044-8309.2010.02000.x 22435846

[bjso70058-bib-0052] Vestergren, S. , & Acar, Y. G. (2023). The dynamic context of transformations through crowds and collective action. Social Research: An International Quarterly, 90(2), 271–292. 10.1353/sor.2023.4901705

[bjso70058-bib-0054] Wright, S. C. , Taylor, D. M. , & Moghaddam, F. M. (1990). Responding to membership in a disadvantaged group: From acceptance to collective protest. Journal of Personality and Social Psychology, 58(6), 994–1003. 10.1037/0022-3514.58.6.994

[bjso70058-bib-0055] Zúñiga, C. , Asún, R. , & Louis, W. (2023). Normative and non‐normative collective action facing repression in a democratic context: A mixed study in a Chilean social movement. Journal of Social and Political Psychology, 11(1), 362–382. 10.5964/jspp.7973

